# Assessment of Chinese rehabilitation assistance system for disabled children

**DOI:** 10.3389/fpubh.2023.1098908

**Published:** 2023-09-14

**Authors:** Zhongyuan Gu, Hong Tan, Haomiao Zhang, Rong Zhou

**Affiliations:** ^1^School of Public Administration, Central South University, Changsha, China; ^2^School of Public Administration, Sichuan University, Chengdu, China; ^3^Rehabilitation Research Center for the Disabled, Hunan Disabled Persons' Federation, Changsha, China

**Keywords:** disabled children, rehabilitation assistance, support system, social support theory, designated rehabilitation institution

## Abstract

Using the social support theory for reference, a subject-object influencing mechanism model of China's rehabilitation assistance system for disabled children is built based on the survey data on 1,698 disabled children in 243 designated rehabilitation institutions in Hu'nan Province as well as the topic of the assessment and optimization of the rehabilitation assistance system for disabled children. The analysis using the structural equation modeling reveals that the inclusive welfare effect of the rehabilitation assistance system for disabled children has emerged, and disabled children receiving free rehabilitation in the designated institutions have achieved good rehabilitation results as a whole, however, disabled children of different age groups have generational differences in the rehabilitation effects, and the preferential aspect of the system needs to be strengthened. Government support, institutional support, and social support have positive non-equilibrium effects in enhancing the rehabilitation effects of disabled children. Institutional support plays a partial mediating role between government support, family support, and disabled children's rehabilitation effects, showing that the current social support system for the rehabilitation assistance of disabled children is experiencing structural, social and kernel changes, to evolve from the traditional closed and disconnected one-way resource support to open, coordinated, and interactive multi-support, and gradually become a comprehensive and efficient interactive support system with families as the foundation, institutions as the main body, and the government as the core.

## Introduction

### Research background

Rehabilitation of disabled children refers to the treatment process of helping disabled children to recover and improve their functional status, reduce dysfunction, and enhance their abilities to take care of themselves in daily life, learn, and socialize through the combination of rehabilitation training, surgery, assistive device adaptation, education, psychological counseling, etc. From the perspective of rehabilitation medicine, childhood is the most economical and effective rehabilitation period with the most far-reaching influence (1). Disabled children are in the stage of growth and development with functions not established; therefore, timely, appropriate, continuous, and effective rehabilitation can not only minimize their physical dysfunction, improve their physical condition, and promote their health and wellbeing (2), but also effectively stimulate their compensatory mechanisms and promote the development of their potential to transform into social advantageous resources, so as to reduce family and social pressure.

Since the 1980s, the Chinese government has been highly valuing the rehabilitation of disabled children and has made improving the welfare and promoting rehabilitation services for disabled children to be a people's livelihood support project. The rehabilitation of disabled children has been included in the outlines of five-year plans from the 8th Five-Year Plan to the 13th Five-Year Plan for the cause of disabled people in China, and the work of rehabilitation of disabled children has been advanced in order. In particular, the work for disabled children has formally entered the stage of system building since the 19th National Congress of the Communist Party of China (CPC) proposed to “develop programs for disabled people and work to provide better rehabilitation services for them”. Acting on the policy requirements to help those most in need, to build a tightly woven safety net, and to build the necessary institutions, the State Council of China introduced China's first special system in the field of rehabilitation of disabled people, i.e., the rehabilitation assistance system for disabled children, in 2018. This system fills in gaps in China's rehabilitation service guarantee system for disabled children and transforms the salvage rehabilitation work for disabled children from project-based operation to institutional guarantee. This system also comprehensively expands the rehabilitation service objects, establishes the rehabilitation assistance principle of doing everything within the capacity to basically realize the salvage of disabled children as much as possible, and comprehensively enhances the rehabilitation welfare of disabled children. According to statistics, from 2018–2021, the annual number of people enjoying rehabilitation assistance for children with disabilities in China is 157,000, 181,000, 237,000, and 363,000, respectively; the total number of disability rehabilitation institutions in China is 9,036, 9,775, 10,440, and 11,260, respectively; the annual number of people in employment in rehabilitation institutions in China is 250,000, 264,000, 295,000, and 318,000, respectively (3–6). All three show a clear trend of rapid increase, and the implementation of the rehabilitation assistance system is particularly effective.

However, despite certain achievements, the system still has many problems in bringing into play to the active role of all subjects involved in the rehabilitation work and meeting the health needs of disabled children. Firstly, there are fewer specialized institutions for the rehabilitation of children with disabilities. Only 24.6% of general hospitals in China have rehabilitation medicine departments, and 20% of provincial general hospitals, 30% of municipal general hospitals and 56% of hospitals below the municipal level do not have early rehabilitation capabilities (7). In addition, rehabilitation institutions for children with disabilities are also of low grade, and there are practical obstacles such as small outdoor space, shortage of funds, and many safety hazards (8), as well as development difficulties such as ambiguous standards for access to rehabilitation institutions, varying levels of rehabilitation, and non-uniform rehabilitation standards (9). Secondly, there are not enough teachers in rehabilitation institutions for children with disabilities. According to the National Bureau of Statistics, <3% of the teaching staff in special education schools in China can provide rehabilitation services for children with disabilities (10), and <8,000 rehabilitation professionals are trained each year, and 70% of them have only a specialist's degree (11), which shows the lack of capacity to train rehabilitation teachers. Thirdly, the coverage of government-implemented rehabilitation programs and social security subsidies provided to children with disabilities is small. According to a survey based in Kunming, China, 48% of children with disabilities have not received free government rehabilitation programs, only 44% of children with disabilities have received free rehabilitation treatment, and only 8% of children with disabilities have received more than 2 sessions of free rehabilitation treatment (7). The vast majority of hospitalization and rehabilitation costs for children with disabilities are not reimbursed through the medical insurance system, and the high cost of rehabilitation medicine can only be borne by the families of children with disabilities. Fourthly, parents of disabled children do not sufficiently understand the rehabilitation policies for disabled children, do not have enough confidence in rehabilitation, and lack knowledge about rehabilitation. Many families of children with disabilities do not have a good understanding of the rehabilitation policy for children with disabilities due to the influence of various factors such as publicity, geography, transportation, and culture. According to a survey, in some areas of China, 78% of the guardians of children with disabilities are not aware of the rehabilitation policy for children with disabilities and the laws and regulations for the protection of the rights and interests of children with disabilities, and 48% of the parents of children with disabilities are not aware of the free rehabilitation program for children with disabilities that the state distributes to local disability federations every year, and do not know how to apply for free rehabilitation places from local disability federations in a timely manner (7).

The problems of the rehabilitation assistance system for disabled children are resulting more from the system's practice logic. The rehabilitation assistance system for disabled children is implemented level by level from the top down in China, which marks a kind of system implementation logic independently promoted by the government, requiring the cooperation and coordination of multiple subjects for exerting the effects. Due to their large differences in the basic conditions, social environment, and cultural atmosphere for the rehabilitation of disabled children, different places are significantly varied and have different problems in the implementation of the rehabilitation assistance system for disabled children. If the cruxes of problems in the implementation of rehabilitation assistance to disabled children are not timely identified and corrected, the implementation effects will not be effectively guaranteed. Therefore, investigating the implementation effects of the rehabilitation assistance system for disabled children in different regions and revealing the roles of different social subjects in the implementation of the said system will be of special practical value to improve the system and comprehensively enhance the implementation performance thereof.

As a macro and systematic concept, the evaluation of the implementation effect of the rehabilitation assistance system for disabled children should include multiple dimensions, such as the source of the rehabilitation system for disabled children, the degree of goal realization, the degree of humanization, the degree of structural soundness, the self-implementation ability, as well as the nature, fairness, simplicity and flexibility of the system. It may also include the degree of compatibility between the rehabilitation system for disabled children and the relevant system, the degree of compatibility with the system environment, and the implementation of the system. However, considering it as a new thing, the system system construction is not yet complete, and some elements are not even established yet, so it cannot be evaluated from a systematic perspective. In addition, the universal focus of the system is to improve the rehabilitation effect of children with disabilities, whether by expanding the scope of rehabilitation assistance, enriching the content of assistance, regularly distributing assistance funds, or providing preferential policies for rehabilitation institutions and strengthening financial security. The fundamental purpose of this system is to improve the rehabilitation effect of children with disabilities. Therefore, starting from the dimension of the realization of the fundamental goal, this paper measures the implementation effect of the rehabilitation assistance system for disabled children with the rehabilitation effect. It is important to note that while the measurement of rehabilitation effects for disabled children is easier to operationalise than the implementation of rehabilitation assistance systems, the assessment of rehabilitation effects is not just a single measure of the traditional physical dimension. The measurement of rehabilitation effects for disabled children should be based on the world-accepted “bio-psycho-social” model of health and disability, which takes into account various aspects such as physical structure, physical functioning, activity and participation, environmental factors and personal factors. Generally speaking, if the physical, psychological and social abilities of disabled children are improved or enhanced as a result of rehabilitation assistance, it means that the system has a positive implementation effect; if the physical, psychological and social abilities of disabled children are not improved as a result of rehabilitation assistance, it means that the implementation effect of the system is negative. Of course, due to the complexity and diversity of the rehabilitation process and outcomes, the rehabilitation effects cannot be measured in terms of good or bad outcomes alone. The specific measurement also requires a more refined study based on the number of different abilities improved, the degree of improvement of each ability, etc., so that the effectiveness of the implementation of the rehabilitation assistance system can be scientifically reflected. The government, to comprehensively promote the implementation of the rehabilitation assistance system for disabled children and ensure its practical effects, should not only improve the system but also pay high attention to the actual situation of disabled children and ensure the effective improvement of the physical indexes of disabled children, so as to maximize the rehabilitation effects of disabled children. Therefore, assessing the implementation effects of the rehabilitation assistance system for disabled children based on the metric of the rehabilitation effects of disabled children is a reasonable strategy.

No studies have been conducted in academia to assess the implementation effects of China's rehabilitation assistance system for disabled children. Most of the studies on the implementation of China's rehabilitation assistance system for disabled children have focused on policy sorting out, policy dilemmas and optimization measures before the issuance of the system as well as the descriptions of implementation status after the issuance. Specifically, related studies can be divided to fall into two stages. The first stage is the building stage of the rehabilitation assistance system for disabled children. Some scholars focused on the studies of fragmented, decentralized, preferential, and project-dependent rehabilitation policies for disabled children before the establishment of the rehabilitation assistance system for disabled children, including, firstly, the sorting out of the rehabilitation policies for disabled children: Zhao by sorting out the rehabilitation policies for disabled children in the past 30 years, summarized the medical rehabilitation model for disabled children of putting prevention first and focusing on early rehabilitation, medical assistance, and salvage treatment (12), Qiao analyzed in detail the implementation paths of rehabilitation welfare for disabled children based on the development history and main achievements of rehabilitation welfare for disabled children and from dimensions such as policy implementation methods, responsibility allocation, and service objects (13); secondly, the descriptions of the implementation status of rehabilitation policies for disabled children and the problems: Fisher, based on a mixed methods research paradigm, found a lack of policy support efforts, a narrow scope of objects eligible for subsidies, insufficient publicity, and a large gap between policy content and concrete implementation through descriptive analysis of survey data from the China Disabled Persons' Federation (CDPF) and a case study approach (14), and Zhao revealed problems with rehabilitation policies for disabled children from dimensions of rehabilitation policy concepts, operation forms, division of responsibilities among competent departments, family and social support systems, and civil society participation (15); thirdly, the proposing of optimization measures for rehabilitation assistance policies for disabled children: Cao et al. proposed improvement measures from dimensions of improving the content of the rehabilitation assistance system for disabled children, establishing a screening and reporting system for disabled children, improving the service capacity of rehabilitation institutions, conducting rehabilitation by multiple subjects, and publicizing rehabilitation policies (16) and Kwok, by studying children with intellectual disabilities in China, put forward that providing targeted assistance for disabled children and strengthening the coordination, communication, and cooperation between the government, families, and NGOs are key initiatives to improve the rehabilitation effects of disabled children (17). The second stage is the study of the implementation of the rehabilitation assistance system for disabled children. Some scholars, based on a developmental policy perspective, believe that a forward-looking social policy should achieve positive social welfare in the dynamic process of social development (18), pointing out that the shift from a “relief-compensation orientation” to a “development-service-orientation” is an important shift in China's welfare policy for children in difficulty (19). The introduction of the rehabilitation assistance system for disabled children has effectively met the rehabilitation needs of disabled children, enhanced their resilience to risks, and given them institutional rights to development, making it an innovative initiative for the healthy development of disabled children (20). However, while acknowledging its effectiveness, some scholars have also shed preliminary light on the problems of the rehabilitation assistance system for disabled children based on the supply and demand dimension. Rehabilitation for disabled children is a long-term process and the costs of surgery, assistive device allocation and rehabilitation training are all relatively high (21). Although the services provided by the rehabilitation assistance for disabled children, such as the allocation of assistive devices and subsidized rehabilitation training, can meet basic rehabilitation needs, some of the surgical and medical rehabilitation items have not yet been covered by medical insurance, and many families of disabled children still bear a relatively heavy cost burden (22). Qi and Wang pointed out that despite China's establishment of a targeted rehabilitation assistance system for disabled children and expansion of the supply of rehabilitation services, there was still a serious gap between supply and demand, which was manifested as exclusion and inclusion errors, resulting in the ineffective implementation of target policies (1). In addition, Sheng et al. by studying 130,290 disabled children and using the logistic regression method, found that there was a gap between the free rehabilitation services provided by the rehabilitation assistance system for disabled children and the rehabilitation needs of disabled children (23).

Overall, the existing research literature has some shortcomings. Firstly, the domestic academia's concern over the rehabilitation of disabled children is far from enough, namely, there are not many scholars who have studied the rehabilitation of disabled children, and there are relatively few study results. Secondly, the points made in the existing research literature have been on a factual and policy basis, which is not wrong and is very necessary, however, stopping there may easily cause a lack of theoretical support for the research conclusions, affecting the rationality of the points and the academic contributions of the research conclusions. Thirdly, the existing research literature has room for further improvement in the research methods. Most of the studies made explorations based on theoretical analysis, normative analysis, case analysis, and simple logistic regression analysis, and lacked quantitative analysis of the rehabilitation assistance system for disabled children. Fourthly, the existing research literature ignores the objective fact that the rehabilitation institutions for disabled children are in an intermediary position in the structure of the action for rehabilitation of disabled children. In China today, the designated rehabilitation institutions for disabled children are service institutions established with the support of disabled persons' federations (DPFs) to specialize in providing treatment training services for the rehabilitation of disabled children, which receive rehabilitation funds from the DPFs or other social entities, receive objects of rehabilitation from the families of disabled children, and play a bridge role in the rehabilitation of disabled children. This is a factor that must be considered in studying the relationships between the rehabilitation effects of and rehabilitation services for disabled children.

In order for the rehabilitation assistance system for disabled children to produce good rehabilitation effects, in this paper, a field survey and empirical research on the rehabilitation assistance system for disabled children have been conducted, with improving the implementation effects of the rehabilitation assistance system for disabled children as the objective, the social support theory as the theoretical basis, and the rehabilitation effects of disabled children as the metric of the system's operation performance. In this paper, “measurement” and “analysis” are combined and structural equation modeling is used to explore from multiple dimensions the action paths and mechanisms of related social subjects' participation in the rehabilitation assistance system for disabled children, dig the deep causes of the unsatisfactory implementation effects of the rehabilitation assistance system for disabled children, and propose pertinent strategies to optimize the effectiveness of the rehabilitation assistance system for disabled children. Therefore, the core questions of this paper are three: Firstly, how effective is the current implementation of the rehabilitation assistance system for children with disabilities in China? Secondly, what is the mechanism through which the support provided by different actors affects the effectiveness of the rehabilitation assistance for children with disabilities? Third, how can the rehabilitation assistance system for children with disabilities be further optimized from the perspective of the support provided by different actors?

### Theoretical framework and research hypotheses

The International Classification of Functioning, Disability and Health (ICF), published by the World Health Organization in 2001, is now widely used in disability statistics and social services as a global and universal standard for terminology, classification and coding for the classification of functioning and disability. The classification is based on a “biopsychosocial” medical model, which states that an individual's functioning in a given domain is the result of the interaction between his or her health status and contextual factors. It sees disability not only as an attribute of the individual, but also as a complex collection of conditions, many of which are caused by environmental factors in the context. Contextual factors refer to all aspects of the external or extrinsic world that form the background to an individual's life, including nature and its features, the man-made natural world, other people with whom the individual has different relationships and roles, attitudes and values, social institutions and services, and policies, rules and laws. These factors are external to the individual, and an environment that facilitates has a positive impact on the individual's performance, ability to move and body structure and function, while an environment that has barriers or lacks enabling factors has a negative impact on the individual's performance, ability to move and body structure and function. Therefore, the solution to disability-related problems needs to focus on the external environment in which people with disabilities live, with the aim of transforming it to enable them to participate in social life and integrate into the social community. Social support, as an important component of the environment, has a corresponding impact on the rehabilitation effectiveness of disabled people. Therefore, in order to further analyse the intrinsic impact of social support on the rehabilitation of disabled children, this paper chooses social support theory as the logical basis and analytical framework.

Originating from the social etiology of mental disorders in the 1970s, the social support theory has rapidly expanded to economics, sociology, pedagogy, etc. owing to its strong explanatory power, to become an important scientific paradigm in these fields. In the social support theory, social support refers to a selective social behavior where a social network helps socially vulnerable groups without compensation through material and spiritual means such as information, laws, finance, and psychology (24). Social support has unique main effect functions (benefit increasing and health care) and buffer functions (buffering and stress reducing) and plays a huge role in the recovery of individuals' physical, psychological, and social abilities. The scientific and integrated combination of different social support elements in a certain way can constitute a social support system for the corresponding socially vulnerable groups. Generally, the broader the social support system for supported objects (i.e., socially vulnerable groups) and the stronger the social support provided by different subjects, the more resources they have to cope with various environmental threats (25), the stronger the unique benefit increasing, health care, buffering, and stress reducing functions of social support, and the correspondingly higher health levels of individuals. The rehabilitation system for disabled children is essentially an integration of the previous fragmented, decentralized, and preferential rehabilitation policies, to build a stable, effective, and sustainable diversified social support network system to provide rehabilitation services for disabled children by linking various social resources, integrating social forces, and scientifically planning the social networks involved in the area of rehabilitation of disabled children. In the system, the government is the system developer and implementer to provide institutional support for disabled children. Rehabilitation institutions are the rehabilitation service providers stipulated by the system to provide the main support for disabled children. Families are the rehabilitation assistance and welfare applicants and daily life caregivers for disabled children, to provide basic support. The three represent the three theoretical subjects of social support, namely, the State, groups, and individuals. Disabled children are at the bottom of the social stratification structure as a vulnerable group, unable to meet their basic needs on their own, being a typical object of social support. Rehabilitation training, surgery, and assistive devices are the means and contents provided to meet the rehabilitation physiological needs of disabled children, being the mediators of social support. In this system, the content and effects of rehabilitation services for disabled children depend on the degree of social support available from the government, rehabilitation institutions, and families of disabled children as well as the stability and effectiveness of the social support system they constitute. The higher the degree of social support available from the government, rehabilitation institutions, and families of disabled children and the more effective their synergetic mechanism, the better the rehabilitation effects of disabled children and the more effective the rehabilitation assistance system for disabled children, and vice versa. In this paper, based on the internal logic of the rehabilitation assistance for disabled children, the social support theory is taken as the theoretical basis for empirical research and the rehabilitation effects of disabled children as the metric of the effects of the rehabilitation assistance system for disabled children to study the comprehensive influence of different subjects of social support on the rehabilitation effects of disabled children and explore the internal operating mechanism of the rehabilitation assistance system for disabled children, so as to find the deep causes of the unsatisfactory implementation effects of the system.

#### Government support and rehabilitation of disabled children

In the rehabilitation support framework for disabled children in China today, the government is the main policy and fund supporter of the rehabilitation cause of disabled children. Based on the definition of the terms “services, institutions and policies for health” and “support and interlinkages” in the ICF main categories of environmental factors and the relevant literature. Government support is effectively the sum of policies, institutions and services provided by the public sector, with government at the center, to address the rehabilitation of children with disabilities, to provide medical rehabilitation and to promote healthy lifestyles (26). In the area of rehabilitation of disabled children, government support is mainly manifested in three action strategies, including institutional guarantee, behavior coordination, and public opinion publicity (27). An institutional guarantee mainly refers to the direct institutional stipulation of the rehabilitation assistance benefits for disabled children and provision of the most direct rehabilitation welfare for disabled children, such as the stipulated standards for rehabilitation assistance funding, the content of rehabilitation assistance, the age eligible for rehabilitation assistance, etc., and this strategy directly determines what free and effective rehabilitation disabled children can enjoy and plays a key role in the rehabilitation effects of disabled children. Behavior coordination refers to that from the perspective of system implementation, the government coordinates DPFs, civil affairs departments, financial departments, health and family planning departments, education departments, human resources and social security departments, etc. to ensure the effective connection among different welfare policies, the convenience of application procedures for rehabilitation assistance, the accuracy of the distribution of rehabilitation assistance benefits, and disabled children's access to rehabilitation services in the most comprehensive, timely, and accurate manner. The purpose of public opinion publicity is to effectively spread the rehabilitation assistance system for disabled children to every family of disabled children and ensure that all disabled children are covered and salvaged as far as possible and timely receive rehabilitation. The above three synergistically influence the rehabilitation effects of disabled children. Therefore, the following research hypothesis is proposed:

H1: Government support positively promotes the rehabilitation effects of disabled children.

#### Support from designated rehabilitation institutions and rehabilitation of disabled children

In the rehabilitation support framework for disabled children in China today, designated rehabilitation institutions are the main service supporter for the rehabilitation of disabled children. A designated rehabilitation institution for disabled children (“rehabilitation institution”) is a medical rehabilitation institution registered according to law to undertake rehabilitation assistance programs for disabled children and provide basic rehabilitation services, such as clinical diagnosis, functional assessment, rehabilitation therapy, function training, and device adaptation for children with various disabilities, which is accredited by a DPF at or above the county level together with education, civil affairs, health, market regulation departments, etc., and signs a rehabilitation service agreement therewith. The common carriers for rehabilitation institutions are mainly hospitals, rehabilitation centers, maternity and childcare hospitals, special education schools and rehabilitation device companies with strong rehabilitation service capacities (28). Staff in rehabilitation institution mainly include health professionals such as doctors, nurses, physiotherapists, occupational therapists, speech therapists, audiologists, prosthetists and orthotists, and medical social workers. As the main provider of rehabilitation services for disabled children, the support capacities of rehabilitation institutions directly determine the levels of rehabilitation sites, rehabilitation equipment, rehabilitation techniques and rehabilitation content accessed by disabled children and have the most direct effect on the rehabilitation effects of disabled children (29). Specifically, rehabilitation institutions mainly provide two types of support for disabled children, namely, hard environment support such as sites and devices, and internal software support such as personnel and techniques. Hooyman pointed out in his study that the environment is not an independent static background, and when individuals want to meet their needs from the environment and adapt to the environment, at least one side needs to make appropriate changes (30). Disabled children are more sensitive to the environment, have limited abilities to adapt to the environment, and cannot make active changes due to the impairment and degradation of their physiological status, sensory functions, and cognitive abilities. Therefore, the professional quality, completeness, and adaptability of the external environment, such as sites, equipment, and space available from the institutions directly determine whether they can meet the needs of disabled children and adapt to them, so as to achieve good rehabilitation effects. Certainly, a perfect and professional external environment is only the foundation for disabled children to obtain good rehabilitation services, and the levels of rehabilitation effects of disabled children depend more on software building such as the professionalism of rehabilitation personnel and the scientificity of rehabilitation techniques (31). Rehabilitation medicine is a typical specialized and refined discipline, involving physical therapy, occupational therapy, speech therapy, prosthetics and orthotics, music therapy, psychotherapy, social therapy, etc. As different rehabilitation therapies provide significantly different personnel and techniques, the treatment methods and contents for disabled children also differ significantly. Whether the institutions have multi-category rehabilitation personnel and refined rehabilitation techniques decides whether disabled children can receive accurate and effective rehabilitation treatment. When disabled children can receive precision treatment by professionals, the treatment effects will be good, and on the contrary, when disabled children receive only cursory treatment by non-professionals, the treatment effects will be poor. Therefore, the following research hypothesis is proposed:

H2: Institutional support positively promotes the rehabilitation effects of disabled children.

#### Family support and rehabilitation of disabled children

In the rehabilitation support framework for disabled children in China today, families of disabled children are the main composite supporter for the rehabilitation of disabled children. Families are basic living units with high internal cohesion and formed based on blood and relationships, and are the main carriers of daily life for disabled children. Based on the definition of the terms “immediate family” and “support and interconnectedness” in the ICF main categories of environmental factors, family support actually refers to the practical material or emotional support, nurturing, protection and assistance in the rehabilitation of disabled children provided by individuals associated with birth, marriage or other culturally recognized immediate family relationships, such as parents and grandparents. Family support is more direct, selfless and sustainable than other forms of support, and is the most fundamental element in the rehabilitation of disabled children (32). Specifically, family support can be roughly divided into three parts: financial support, life care, and emotional support (33), which influence the rehabilitation effects of disabled children from different dimensions. Through direct financial help and material support, financial support provides disabled children with funds for daily life, rehabilitation training, surgery, and purchase of family rehabilitation assistive devices, daily nourishment, etc. The amount of financial support directly determines the quantity and quality of rehabilitation services purchased by families and plays a fundamental role in the rehabilitation of disabled children. From the perspective of family daily rehabilitation, life care refers to families' provision of daily, standardized, and continuous rehabilitation training to disabled children by providing residence, food, daily care, safety protection, companionship, daily rehabilitation, etc. The more knowledgeable and skilled the family members are in daily rehabilitation, the more effective the rehabilitation services for disabled children at home. Through positive emotional reinforcement, family relationship adjustment, reasonable stress relief, etc., emotional support reduces the internal inferiority complex, loneliness, and social disengagement feeling of disabled children, strengthens their resistance to mental stress, self-confidence, and optimism, and plays a key role in mental and emotional recovery. The three parts have a combined influence on the rehabilitation of disabled children based on the medium of families. The more family support, the more resources and advantages disabled children have for rehabilitation treatment, and the more rehabilitation effects they receive. Therefore, the following research hypothesis is proposed:

H3: Family support positively promotes the rehabilitation effects of disabled children.

#### Mediating role of rehabilitation institution support

In the previous project-based social support system for the rehabilitation of disabled children, the government directly provided financial support and purchased assistive devices for disabled children in some archived poverty-stricken households and low-income households by mainly relying on specific service projects, such as the Ministry of Civil Affairs' “Tomorrow Plan” and “Rebirth Action,” and the CDPF's “Public Welfare Lotteries for Rehabilitation” and “Colorful Dream Action Plan” (14); rehabilitation institutions operated in reliance on charges for daily rehabilitation and assistive devices of disabled children in some wealthy families. Families of disabled children provided life care and rehabilitation services for disabled children solely according to their subjective experience. The subjects of support engaged with the object of support, i.e., disabled children, through the typical “sending—receiving” one-way delivery pathway, and the flow of support resources between different subjects showed an apparent one-way characteristic, resulting in clear boundaries between the subjects and between the subjects and the object (34). In the new rehabilitation assistance system for disabled children, the previous status of disconnection between the subjects of assistance has been changed, where the government starts to become the supporter, coordinator and supervisor, families of disabled children become the cooperator, beneficiary and supervisor, and rehabilitation institutions become the functional medium for uniting the subjects and the platform and base for the joint efforts to be effective. The three sides are closely connected to jointly support rehabilitation (1). Specifically, rehabilitation institutions, on the basis of ensuring their direct effect in the rehabilitation, become the core platform for the subjects to gather social support and launch rehabilitation assistance support activities. not only do they need to undertake the rehabilitation assistance functions commissioned by the government for disabled children, become the direct users of rehabilitation funds from the government, and accept the government's audit and supervision as well as daily work guidance (11), but they also need to assist families in applying for free rehabilitation policies, meet the individualized expectations of families with disabled children, satisfy their counseling, professional, emotional, financial and service needs (10), provide different types of rehabilitation services for disabled children in different families, and receive the parents' supervision over the government's funds for free rehabilitation. On this basis, rehabilitation institutions can then provide rehabilitation training, surgical correction, assistive device adaptation, and other services according to their actual situations, such as rehabilitation facilities, site area, and staff strength. This way, the government, institutions, and families can form an integrated collaboration system, to comprehensively influence the rehabilitation of disabled children. In fact, the theoretical relationship between the three has been discussed in the academic field for a long time. Yao pointed out that rehabilitation institutions for people with disabilities not only undertake rehabilitation medical training, community rehabilitation technical guidance, rehabilitation information consultation, rehabilitation research and disability prevention arranged by the government, but also assist families of people with disabilities in clinical diagnosis, functional assessment, formulation of rehabilitation plans and implementation of rehabilitation treatment, etc. It is comprehensive windows and technical resource centers for the rehabilitation of people with disabilities in their areas, effectively linking families and the government (35). Shu also believes that an efficient social support system for children with disabilities should be built on the basis of giving full play to the government's functions of supporting rehabilitation institutions with resources, legal regulation and daily guidance, and actively urging families to monitor and inquire about rehabilitation institutions (36). Therefore, the following research hypothesis is proposed:

H4: Institutional support plays a mediating role between family support or government support and the rehabilitation effects of disabled children.

## Methodology

### Model selection

To further verify the above research hypotheses, the structural equation modeling (SEM) is selected in this paper to measure the relationships between variables, with the specific model equations as follows:


(1) $$\begin{array}{l} \text{Structural equation}:\text{ S}=\Gamma L+\zeta \end{array}$$

(2) $$\begin{array}{l} \text{Endogenous variable measurement equation}:\text{ Y}=\text{Λ}yS+\varepsilon \end{array}$$

(3) $$\begin{array}{l} \text{Exogenous variable measurement equation}:\text{ X}=\text{Λ}xL+\delta \end{array}$$

Where, Equation (1) is a structural model representing the relationships between the exogenous latent variable and the endogenous latent variable, with S representing the endogenous latent variable, i.e., the rehabilitation effects of disabled children, L representing the three exogenous latent variables (government support, institutional support, and family support), Γ representing the path coefficient (the influence of the exogenous latent variables on the endogenous latent variable), and ζ representing the residual term (reflecting the unexplained part of the equation).

Equations (2) and (3) are measurement models that connect the latent variables and the observed variables, where, Y represents the observed variable under the endogenous latent variable, X represents the observed variable under the exogenous latent variable, Λ y and Λ x represent the factor loading matrix of the observed variable under the endogenous latent variable and that of the observed variable under the exogenous latent variable, respectively, ε represents the measurement error of the observed variable under the endogenous latent variable, and δ represents the measurement error under the exogenous latent variable.

### Variable selection and measurement

#### Dependent variable

Rehabilitation effects of disabled children. The rehabilitation effects of disabled children refer to the specific situation of functional improvement of disabled children after various rehabilitation activities and represent an important metric of the implementation situation and effects of the rehabilitation assistance system for disabled children. According to the existing studies, there are various scales to measure the rehabilitation effects, such as the Nottingham Health Profile (NHP) (37), the International Classification of Functioning, International Classification of Functioning, Disability and Health (ICF), the Pediatric Quality of Life Inventory (PedsQL) (38), and the World Health Organization Quality of Life (WHOQOL-100) (39). The Nottingham Health Scale, made by the Faculty of Social Medicine at the University of Nottingham in the UK in the early 1980s, consists of six dimensions of physical activity, energy, pain, sleep, social connectedness and emotional response and seven dimensions of work, caring for the family, social life, family life, sexual life, hobbies and interests, and vacation. The Quality of Life Scale for Children and Adolescents is a self-assessment scale for measuring the quality of life of primary and secondary school students aged 7–18 years old, which was developed by the Department of Child and Adolescent Health, Tongji Medical College, Huazhong University of Science and Technology in 2000, including self-satisfaction, teacher-student relationship, somatic sensation, peer relationship, parent-child relationship, motor ability, learning ability and attitude, self-concept, negative emotion, homework attitude, activity opportunity, and convenience of life. and other 13 dimensions of measurement indicators. The International Classification of Functioning, Disability and Health is a health scale published by the World Health Organization to measure a number of aspects of human body structure, physical function, activity and participation, environmental factors and personal factors. The WHO Quality of Life Scale is an international scale developed by the World Health Organization to measure the quality of life of individuals in relation to their health, including indicators of physical health, psychological status, independence, social relationships, personal beliefs, and relationships with neighbors. Although these scales are all scales about human physical and mental health, their measurement objectives and functions are quite different, and their internal indicators do not fit the physical and mental conditions of children with various types of disabilities, so they cannot be directly used to measure the rehabilitation effects of children with disabilities. However, the dimensions of physical ability, mental ability, social ability, and emotional ability contained in these scales (40) reflect the common requirements of human physical and mental health and can be used to design rehabilitation effect assessment dimensions for children with disabilities, so this paper borrows these four dimensions to measure the rehabilitation effect of children with disabilities. The questionnaire used a five-point Likert scale of “very little improvement, relatively little improvement, fair, relatively much improvement and much improvement,” and parents were asked about the degree of improvement in their children's self-care ability, psychological state, verbal communication ability, emotion perception ability, and overall condition after rehabilitation treatment.

#### Independent variable

##### Government support

Government support refers to the sum of various systems and services provided by public departments with the government as the axis for socially vulnerable groups and is an important measure of vulnerable groups' access to support resources. Government support was mainly measured from the dimensions of institutional guarantee, behavior coordination, and public opinion support based on the various preferential policies provided for the rehabilitation of disabled children in the Implementation Opinions on the Establishment of Rehabilitation Assistance System for Disabled Children in Hu'nan Province and Xu's definition of government support for children (27). The dimension of institutional guarantee was measured according to the rationality of the content of rehabilitation assistance and the scientificity of the setting of rehabilitation assistance funds. The dimension of behavior coordination was measured according to the convenience of rehabilitation assistance procedures and the satisfaction with the help provided by other government departments (such as human resources and social security departments and education departments) for the rehabilitation of disabled children. The dimension of public opinion support was measured according to the effectiveness of publicity methods. The answer to the question item was measured on a five-point Likert scale.

##### Family support

Family support refers to the help provided by other family members for the vulnerable members. In the light of disabled children's psychological similarities to preschool children and physiological similarities to the disabled older adult, in this study, based on the measurement of family support for preschool children by Yue and Zhang and the measurement of family support for the disabled older adult by Chen et al. (41, 42), family support for disabled children was mainly measured at three levels: financial support, life care, and spiritual comfort in conjunction with the special characteristics of disabled children. The specific measurement items are as follows: Apart from the government subsidy, are you willing to spend more on your child's rehabilitation each month? How well do you and your family members know about rehabilitation? How confident are you in your child's rehabilitation? The answer to the question item was measured on a five-point Likert scale.

#### Mediating variable

##### Rehabilitation institution support

Rehabilitation institution support refers to the sum of various services provided by medical rehabilitation institutions such as hospitals, rehabilitation centers, maternity and childcare hospitals, and special education schools for the rehabilitation population, being an important metric of the institution-based rehabilitation level. In the light of the special requirements of disabled children for institution-based rehabilitation, in this study, after synthesizing the detailed indicators for designated rehabilitation institutions in the Notice on the Exit and Access of Designated Rehabilitation Institutions for Disabled Children as well as the evaluation dimensions put forward by Mi et al. for rehabilitation institutions (43), two dimensions of rehabilitation institution support were mainly included: hard environment support, such as sites and devices, and internal software support, such as personnel and techniques. The variables measured for hard environment support included the satisfaction with the comprehensive environment of the rehabilitation institution and the completeness of rehabilitation equipment, both of which were ordinal variables. The variables measured for internal software support included the professionalism of rehabilitation personnel, the advanced nature of rehabilitation techniques, and the rationality of rehabilitation content, all of which were ordinal variables. The answer to the question item was measured on a five-point Likert scale. See Table 1 for the descriptive statistics of the variables.

**Table 1:** Meanings and descriptive statistical analysis of variables.

**Variable**	**Meaning**	**Variable code**	**Variable type**	**Variable assignment**	**Mean**	**Standard deviation**
Rehabilitation effects	Self-care ability	eff1	Ordinal	1 = very little improvement, 2 = relatively little improvement, 3 = fair, 4 = relatively much improvement, 5 = much improvement	3.21	1.144
	Psychological state	eff2	Ordinal	1 = very little improvement, 2 = relatively little improvement, 3 = fair, 4 = relatively much improvement, 5 = much improvement	2.97	1.073
	Verbal communication ability	eff3	Ordinal	1 = very little improvement, 2 = relatively little improvement, 3 = fair, 4 = relatively much improvement, 5 = much improvement	3.01	1.222
	Emotion perception ability	eff4	Ordinal	1 = very little improvement, 2 = relatively little improvement, 3 = fair, 4 = relatively much improvement, 5 = much improvement	3.31	1.01
	Overall improvement	eff5	Ordinal	1 = very little improvement, 2 = relatively little improvement, 3 = fair, 4 = relatively much improvement, 5 = much improvement	3.4	1
Government support	Rationality of the content of assistance	pol1	Ordinal	1 = very dissatisfied, 2 = relatively dissatisfied, 3 = average, 4 = relatively satisfied, 5 = very satisfied	3.6	0.892
	Scientificity of the setting of assistance funds	pol2	Ordinal	1 = very dissatisfied, 2 = relatively dissatisfied, 3 = average, 4 = relatively satisfied, 5 = very satisfied	3.46	0.935
	Convenience of assistance procedures	pol3	Ordinal	1 = very dissatisfied, 2 = relatively dissatisfied, 3 = average, 4 = relatively satisfied, 5 = very satisfied	3.67	0.868
	Coordination of cooperation among departments	pol4	Ordinal	1 = very dissatisfied, 2 = relatively dissatisfied, 3 = average, 4 = relatively satisfied, 5 = very satisfied	3.38	1.009
	Effectiveness of publicity methods	pol5	Ordinal	1 = very dissatisfied, 2 = relatively dissatisfied, 3 = average, 4 = relatively satisfied, 5 = very satisfied	3.56	0.871
Family support	Spending on treatment	fam1	Ordinal	1 = less willing, 2 = more reluctant, 3 = average, 4 = more willing, 5 = very willing	3.71	0.959
	Knowledge of rehabilitation	fam2	Ordinal	1 = very blurred, 2 = relatively blurred, 3 = fair, 4 = relatively clear, 5 = very clear	3.37	0.763
	Confidence in rehabilitation	fam3	Ordinal	1 = very much not, 2 = relatively not, 3 = average, 4 = relatively yes, 5 = very much	3.36	0.876
Institutional support	Comprehensive environment	mec1	Ordinal	1 = very dissatisfied, 2 = relatively dissatisfied, 3 = average, 4 = relatively satisfied, 5 = very satisfied	3.81	0.836
	Completeness of rehabilitation equipment	mec2	Ordinal	1 = very dissatisfied, 2 = relatively dissatisfied, 3 = average, 4 = relatively satisfied, 5 = very satisfied	4.19	0.734
	Professionalism of rehabilitation personnel	mec3	Ordinal	1 = very dissatisfied, 2 = relatively dissatisfied, 3 = average, 4 = relatively satisfied, 5 = very satisfied	3.82	0.923
	Advanced nature of rehabilitation techniques	mec4	Ordinal	1 = very dissatisfied, 2 = relatively dissatisfied, 3 = average, 4 = relatively satisfied, 5 = very satisfied	3.94	0.803
	Rationality of rehabilitation content	mec5	Ordinal	1 = very dissatisfied, 2 = relatively dissatisfied, 3 = average, 4 = relatively satisfied, 5 = very satisfied	3.92	0.807

### Data source

This study was based on the results of a questionnaire survey conducted in 243 designated rehabilitation institutions in 14 prefectural-level cities in Hu'nan Province from June to December 2020, with the respondents mainly being the parents of disabled children who have received rehabilitation in the designated rehabilitation institutions. Considering that the number of people who have received rehabilitation in the designated institutions in each prefectural-level city was different, in order to ensure the representativeness of the survey and the reasonableness of the samples, the equal probability sampling method was mainly adopted in the survey. Firstly, the number of designated rehabilitation institutions for disabled children in each city of the province (53 in Changsha, 10 in Zhuzhou, 12 in Xiangtan, 17 in Hengyang, 16 in Shaoyang, 32 in Yueyang, 14 in Changde, 9 in Zhangjiajie, 15 in Yiyang, 15 in Chenzhou, 11 in Yongzhou, 15 in Huaihua, 13 in Loudi, and 11 in Xiangxi) was used as the sampling basis, the theoretical number of questionnaires to be distributed in each county and city was determined based on eight questionnaires per institution (424 in Changsha, 80 in Zhuzhou, 96 in Xiangtan, 136 in Hengyang, 128 in Shaoyang, 256 in Yueyang, 112 in Changde, 72 in Zhangjiajie, 120 in Yiyang, 120 in Chenzhou, 88 in Yongzhou, 120 in Huaihua, 104 in Loudi, and 88 in Xiangxi), then, with the assistance of the Hu'nan Disabled Persons' Federation, surveyors were dispatched to each designated rehabilitation institution in the province to conduct a structured questionnaire survey, and finally, 1,944 questionnaires were distributed in total. After invalid questionnaires were eliminated, a total of 1,698 valid questionnaires were recovered, with a valid recovery rate of 87.4%.

## Result

### Descriptive analysis

#### Sample characteristics

As shown in Table 2, among the survey samples, the proportions of disabled children aged 0–3, 3–6, and 6–14 were 25.7, 60.5, and 13.8%, respectively, in line with the objective reality that currently, cities in Hu'nan Province mainly provide assistance to disabled children aged 0–6 and only some cities provide rehabilitation assistance to disabled children aged above 6. It should be noted that, given the developmental trajectory of the child, disabled children between the ages of 0 and 3 years are primarily trained in habilitation, which focuses on helping them to develop their functions in order to adapt to daily life, while disabled children after the age of 3 years are primarily trained in specialized rehabilitation, which focuses on restoring and improving the individual's function and autonomy. There were 892 (52.5%) boys and 806 (47.5%) girls, respectively, showing that the sex ratio was reasonable. There were 411 (24.2%) disabled children with urban household registration and 1,287 (75.8%) disabled children with rural household registration, respectively, in line with the current basic feature that the number of disabled children in rural areas is larger than that of disabled children in urban areas. From the perspective of self-care, 49.1% of disabled children could barely take care of themselves, 31% could partially do so, and only 19.8% could basically do so, showing the current situation that disabled children in Hu'nan Province have serious functional impairments. Furthermore, most of the disabled children in the survey had Class 1 (21.6%), Class 2 (28.6%), and undetermined class (34.6%) of disabilities, and the proportions of disabled children with Class 3 and Class 4 disabilities were only 9.1 and 6.1%, respectively. The proportions of children with visual, hearing, speech, physical, intellectual, mental and multiple disabilities were 3.7, 19.1, 17.8, 5.4, 19.8, 16.5, and 17.7%, respectively.

**Table 2:** Sample data characteristics.

**Variable**	**Type**	**Frequency**	**Proportion (%)**	**Variable**	**Type**	**Frequency**	**Proportion (%)**
Gender	Male	892	52.5	Household registration	Urban	411	24.2
	Female	806	47.5		Rural	1,287	75.8
Age	0–3 years old	437	25.7	Self-care	Totally incompetent	448	26.4
	3–6 years old	1,027	60.5		Largely incompetent	388	22.9
	6–14 years old	234	13.8		Partly competent	526	31
Type of disability	Visual disability	62	3.7		Largely competent	266	15.7
	Hearing disability	324	19.1		Totally competent	70	4.1
	Speech disability	302	17.8	Class of disability	Class I	366	21.6
	Physical disability	92	5.4		Class II	485	28.6
	Intellectual disability	337	19.8		Class III	155	9.1
	Mental disability	280	16.5		Class IV	104	6.1
	Multiple disabilities	301	17.7		Undetermined	588	34.6

#### Descriptive statistics of policy implementation effects

The rehabilitation effects of disabled children are the most direct and effective metric of the implementation effects of the rehabilitation assistance system for disabled children. The better the rehabilitation effects achieved by disabled children receiving free rehabilitation training in designated institutions, the better the implementation effects of the rehabilitation assistance system for disabled children. Therefore, to better measure the rehabilitation effects of disabled children and the implementation effects of the system, in this study, efforts were made to attempt to further explore the improvement of disabled children in physical, psychological, communication, perception and overall abilities and then the five scores were summed to obtain the comprehensive improvement score of disabled children, to comprehensively highlight the rehabilitation effects of disabled children. One-way ANOVA was used to further analyze the differences in rehabilitation effects among different age groups. The results are shown in Table 3. The mean of the comprehensive improvement scores of rehabilitation effects of disabled children ranges from 15.133 to 16.328, showing a good overall level of improvement and the emergence of the inclusive welfare effect of the rehabilitation assistance system for disabled children. Furthermore, since the system was issued, disabled children aged 3–6 have achieved the maximum rehabilitation effects, followed by disabled children aged 6–14, and disabled children aged 0–3 have had the minimum improvement effects, showing that the rehabilitation effects of disabled children have had an inverted U-shaped development trend with the increase of age. According to the one-way ANOVA results, the differences in each rehabilitation effect are statistically significant across age groups (all *P* < 0.05), indicating significant generational differences in the rehabilitation effects of different age groups. Also, it is worth noting that, except for disabled children aged 3–6 who score more than 3 in the improvement of all abilities, disabled children aged 0–3 score <3 in the improvement of self-care ability, psychological development ability, and verbal communication ability, which is poor. Disabled children aged 6–14 also score <3 in the improvement of psychological development ability and verbal communication ability, which is at average and below. The above result reflects, to some extent, that the system lacks specificity in the specific implementation and fails to effectively respond to the rehabilitation content of disabled children of different age groups, and the system's preferential aspect needs to be strengthened.

**Table 3:** Statistics and difference analysis of rehabilitation effects of disabled children of different age groups.

**Variable**	**0–3 years old**		**3–6 years old**		**6–14 years old**		**ANOVA result**	
	**Mean**	**Standard deviation**	**Mean**	**Standard deviation**	**Mean**	**Standard deviation**	**F**	**P**
Comprehensive improvement score	15.133	4.837	16.328	4.658	15.449	4.999	10.918	0.000
Self-care ability	2.9	1.155	3.35	1.109	3.21	1.167	23.951	0.000
Psychological development ability	2.9	1.052	3.02	1.073	2.85	1.101	3.579	0.028
Verbal communication ability	2.82	1.198	3.12	1.221	2.9	1.221	10.172	0.000
Emotion perception ability	3.2	1.031	3.38	0.978	3.18	1.076	7.277	0.001
Overall ability	3.31	1.029	3.46	0.971	3.32	1.054	4.714	0.009

### Prerequisite tests

#### Exploratory factor analysis

To measure whether the scale elements of this questionnaire and the concepts in the theoretical model hypotheses can match one another, in this study, efforts were made to attempt to preliminarily test the samples through exploratory factor analysis. Firstly, a factor analysis suitability test was conducted, and the results showed that the KMO value was 0.938 and the value of Bartlett's test of sphericity was 21,236.6 (*p* < 0.001), showing that the sample data was suitable for factor analysis. Then, the principal component analysis method and the varimax rotation method were adopted to solve the common factors, a total of four common factors were extracted based on the principle of eigenvalues greater than one, all common factor loads were >0.65, and the cumulative variance explained reached 74.505%, showing that the extracted four common factors could effectively cover the basic information on each measurement indicator and had a strong generalization ability. Also, seen from the structural distribution of the common factors, the measurement indicators were effectively concentrated in their respective dimensions, showing that the extracted common factors effectively matched the conceptual dimensions assumed in the above theoretical model. See Table 4 for details.

**Table 4:** Factor rotation load matrix and total variance explained.

**Variable**	**Rehabilitation effect**	**Government support**	**Institutional support**	**Family support**
pol1		0.713		
pol2		0.847		
pol3		0.732		
pol4		0.822		
pol5		0.854		
mec1			0.668	
mec2			0.818	
mec3			0.778	
mec4			0.813	
mec5			0.759	
fam1				0.692
fam2				0.861
fam3				0.875
eff1	0.84			
eff2	0.813			
eff3	0.836			
eff4	0.82			
eff5	0.809			
% of variance explained by each factor	21.818%	20.643%	20.055%	11.99%
Cumulative % of variance explained	21.818%	42.46%	62.515%	74.505%

#### Confirmatory factor analysis

The confirmatory factor analysis can effectively confirm the quality of fitting between each scale dimension and the data, and if the quality is good, it means that the scale has good reliability and validity; therefore, in this study, the reliability and validity were further tested through the confirmatory factor analysis. The results showed that in terms of reliability, the factor loadings of the observed variables under the four latent variables were greater than the specified threshold of 0.5 (44), the Cronbach Alpha coefficients of the four dimensions were 0.911, 0.904, 0.777, and 0.923, respectively, which were >0.6, and the composite reliability ranged from 0.794 to 0.925, which exceeded the acceptable threshold of 0.7, showing that each dimension had good reliability and had consistency, stability, and aggregation. In terms of validity, the KMO value of the overall scale was first tested to be 0.938, which was >0.7, and the value of Bartlett's test of sphericity was 21,236.6 (*p* < 0.001), showing that the survey data had good construct validity as a whole; then, the AVE and $\sqrt{AVE}$ values were used to measure the convergent validity of the scale, and the results showed that the AVE value of each latent variable was greater than the acceptable level of 0.5, and the $\sqrt{AVE}$ value of each latent variable was greater than the correlation coefficient with other latent variables, showing that good convergent validity existed within each dimension and good discriminant validity existed between the dimensions. See Tables 5, 6 for details.

**Table 5:** Analysis of reliability and convergent validity of variables.

**Dimension**	**Item**	**Factor loading**	**Internal consistency reliability**	**Composite reliability**	**Convergent validity**
		**Std**	**Cronbach, s**α	**CR**	**AVE**
Government support	pol1	0.785	0.911	0.913	0.678
	pol2	0.868			
	pol3	0.737			
	pol4	0.813			
	pol5	0.903			
Institutional support	mec1	0.698	0.904	0.908	0.665
	mec2	0.816			
	mec3	0.769			
	mec4	0.915			
	mec5	0.864			
Family support	fam1	0.607	0.777	0.794	0.567
	fam2	0.848			
	fam3	0.783			
Rehabilitation effects	eff1	0.835	0.923	0.925	0.713
	eff2	0.838			
	eff3	0.843			
	eff4	0.834			
	eff5	0.87			

**Table 6:** Analysis results of discriminant validity.

	**Government support**	**Institutional support**	**Family support**	**Rehabilitation effects**
Government support	**0.823**			
Institutional support	0.676	**0.815**		
Family support	0.279	0.322	**0.753**	
Rehabilitation effects	0.487	0.605	0.399	**0.823**

Diagonal bold font indicates the square root of AVE. The lower triangle presents Pearson's correlation coefficients between dimensions.

### Model goodness-of-fit test

To further verify the research hypotheses, measure the relationships between the three dimensions, and examine model fitting, in this paper, the goodness of fit of the model was tested using AMOS 23.0 software. Generally, when the parsimonious fit indices (PGFI, PNFI) are >0.5 (45), it means that the model has good goodness of fit. When the difference indices (RMR, RMSEA) of the absolute fit indices are <0.08 (45, 46) and the similarity indices (GFI, AGFI) thereof are >0.9 (47), it means that the model has good goodness of fit. When the incremental fitness indices (NFI, IFI, TLI, CFI) are >0.9 and approach 1 (46–48), it means a better fit of the data and the model. After testing, the model had PGFI = 0.724, PNFI = 0.818, RMR = 0.043, RMSEA = 0.049, GFI = 0.96, AGFI = 0.947, NFI = 0.97, IFI = 0.976, TLI = 0.971, and CFI = 0.976. All the fit indices met the corresponding criteria, showing that the model had good fitting overall. See Table 7 for details.

**Table 7:** Model goodness-of-fit test results.

**Evaluation index**		**Recommended value**	**Model**	**Result**
Parsimonious fit indices	PGFI	>0.5	0.724	Good
	PNFI	>0.5	0.818	Good
Absolute fit indices	RMR	< 0.08	0.043	Good
	RMSEA	< 0.08	0.049	Good
	GFI	>0.9	0.96	Good
	AGFI	>0.9	0.947	Good
Incremental fitness indices	NFI	>0.9	0.97	Good
	IFI	>0.9	0.976	Good
	TLI	>0.9	0.971	Good
	CFI	>0.9	0.976	Good

### Model estimation results and analysis

**Figure 1:**
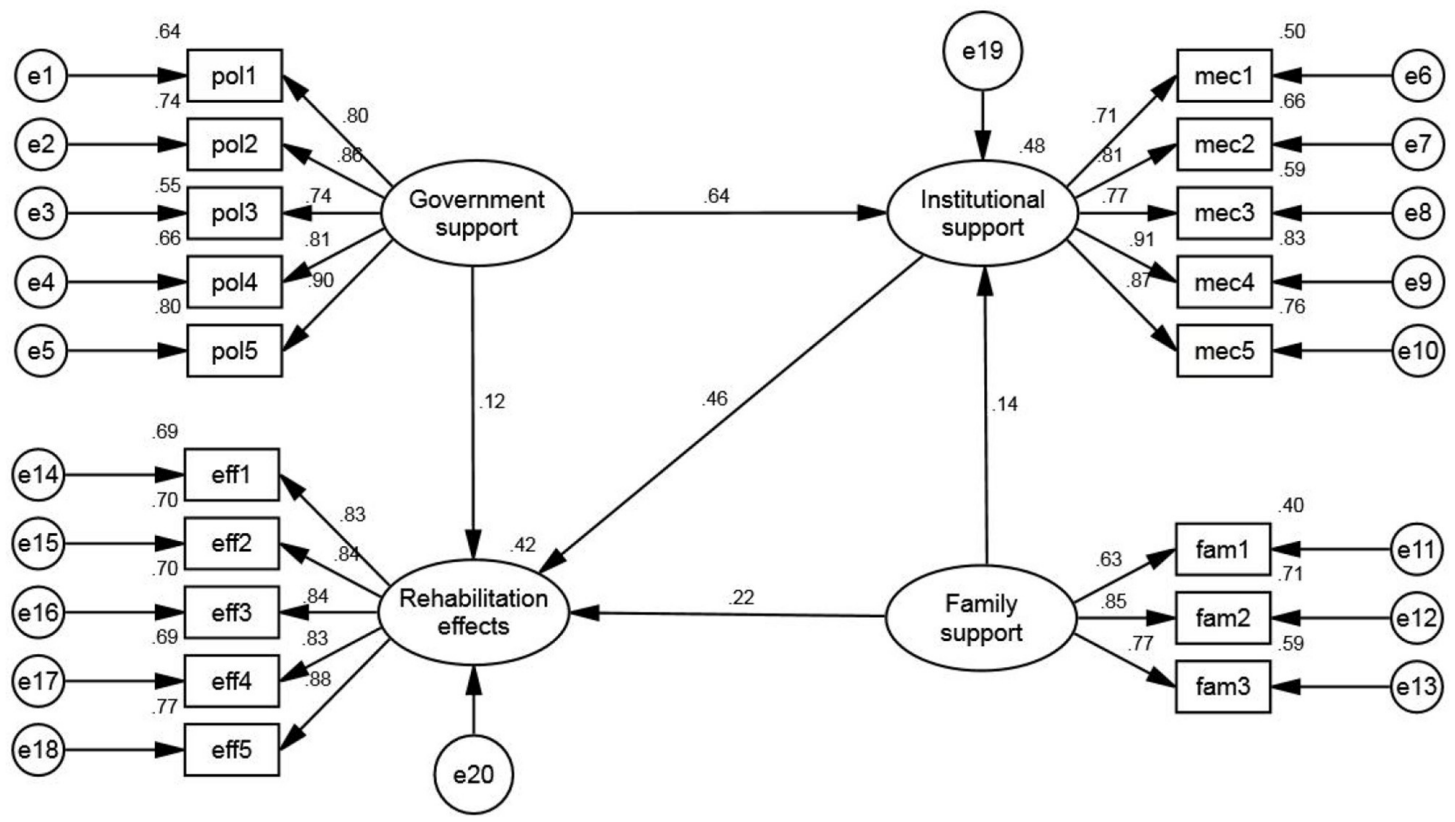
Structural equation modeling diagram.

After fitting, the detailed results of the empirical analysis of the model are shown in Figure 1 and Table 8.

**Table 8:** Influence of government, institutional and family support on rehabilitation effects.

**Path**	**Point estimation**			**Bootstrapping**			
		**Product of coefficients**		**Bias-corrected 95%CI**		**Percentile 95%CI**	
		**SE**	**Z**	**Lower**	**Upper**	**Lower**	**Upper**
**Direct effect**							
Institutional support ← Family support	0.144	0.023	6.261	0.097	0.190	0.098	0.190
Institutional support ← Government support	0.636	0.022	28.909	0.587	0.674	0.589	0.676
Rehabilitation effects ← Family support	0.219	0.027	8.111	0.159	0.272	0.166	0.277
Rehabilitation effects ← Government support	0.118	0.033	3.576	0.059	0.187	0.054	0.183
Rehabilitation effects ← Institutional support	0.455	0.032	14.219	0.391	0.515	0.392	0.516
**Indirect effect**							
Rehabilitation effects ← Family support	0.066	0.011	6.000	0.043	0.088	0.043	0.088
Rehabilitation effects ← Government support	0.289	0.023	12.565	0.247	0.338	0.246	0.336
**Total effect**							
Institutional support ← Family support	0.144	0.023	6.261	0.097	0.190	0.098	0.190
Institutional support ← Government support	0.636	0.022	28.909	0.587	0.674	0.589	0.676
Rehabilitation effects ← Family support	0.285	0.029	9.828	0.229	0.343	0.231	0.345
Rehabilitation effects ← Government support	0.407	0.026	15.654	0.360	0.459	0.357	0.456
Rehabilitation effects ← Institutional support	0.455	0.032	14.219	0.391	0.515	0.392	0.516

The 1,000 bootstrap samples. The effect is significant if the confidence interval does not contain a value of 0.

#### Direct effect analysis

According to Table 8 and Figure 1, in the results of direct effect analysis, government support, institutional support, and family support were all found to have significant positive effects on the rehabilitation effects of disabled children, with effects of 0.118, 0.455 and 0.219, respectively, showing that, with other conditions unchanged, the greater the total amount of government support, institutional support and family support enjoyed by disabled children, the greater the promotion effect on the rehabilitation effects of disabled children, and research hypotheses H1, H2, and H3 hold. In addition, the direct effect of government support and family support on institutional support is 0.636 and 0.144, respectively, being statistically significant, showing that government support and family support also have significant positive effects on institutional support.

#### Mediating effect analysis

Common mediating effect testing methods include stepwise regression, Sobel test, difference coefficient test, and bootstrap method. Among them, the bootstrap method does not only allow variables to contain measurement errors but also can include all data information and reduce the loss of information, playing a unique role in mediating effect testing. Therefore, the bootstrap method was adopted in this study to analyze the significance of the mediating effect. It was estimated using the bootstrapping procedure of Amos 23.0 with 1,000 times of resampling at the 95% confidence interval and based on the maximum likelihood method. Generally, when the absolute value of Z is ≥1.96 or when neither the bias-corrected confidence interval nor the percentile confidence interval contains 0 at the 95% confidence level, it means that the indirect effect is significant. According to the results, the indirect effect of government support through institutional support on the rehabilitation effects of disabled children is 0.289 with a z-score of 12.565, which is >1.96, and its bias-corrected and percentile confidence intervals do not contain 0, showing a significant mediating effect of institutional support in the path of “government support—institutional support—rehabilitation effects,” playing a partial mediating role. The indirect effect of family support through institutional support on the rehabilitation effects of disabled children is 0.066 with a z-score of 6, which is >1.96, and its bias-corrected and percentile confidence intervals do not contain 0, showing a significant mediating effect of institutional support in the path of “family support—institutional support—rehabilitation effects,” playing a partial mediating role. Therefore, research hypothesis H4 holds.

#### Total effect analysis

According to the results of the total effect analysis, the total effects of family support and government support on institutional support are consistent with the direct effects thereof, and government support, institutional support, and family support have significant positive, diversified, and non-equilibrium effects on promoting the rehabilitation of disabled children, among which, the promotion effect of institutional support is the largest, followed by government support, and the promotion effect of family support is the smallest. Specifically, the total effect of government support on the rehabilitation effects of disabled children is 0.407, mainly consisting of the direct effect of “government support—rehabilitation effects” (0.118) and the indirect effect of “government support—institutional support—rehabilitation effects” (0.289), separately accounting for 28.99 and 71.01%. The effect of institutional support on the rehabilitation effects of disabled children is a single direct effect, with a total effect of 0.455. The total effect of family support on the rehabilitation effects of disabled children is 0.285, including the direct effect of “family support—rehabilitation effects” (0.219) and the indirect effect of “family support—institutional support—rehabilitation effects” (0.066), accounting for 76.84 and 23.16% of the total effect of family support, respectively.

## Discussion, conclusion and policy recommendations

### Discussion and conclusion

In this study, based on the survey data on 1,698 disabled children in 243 designated rehabilitation institutions in Hu'nan Province, the structural equation modeling and the bootstrap method are adopted to study the influence paths and effects of government support, institutional support, and family support on the rehabilitation effects of disabled children. The results show that:

The inclusive welfare effect of the rehabilitation assistance system for disabled children has emerged, and disabled children receiving free rehabilitation in the designated institutions achieved good rehabilitation results as a whole, however, disabled children of different age groups have significant generational differences in the rehabilitation effects, the rehabilitation effects of disabled children have an inverted *U*-shaped development trend with the increase of age, and the preferential aspect of the system needs to be strengthened. Of course, it is worth stating that there is a large difference in the proportion of rural and non-rural samples in this study. When the 1,698 survey samples were counted, it was found that there were only 411 children with disabilities from urban household registration, accounting for only 24.2% of the sample, while there were as many as 1,287 children with disabilities from rural household registration, accounting for 75.8% of the sample. This situation seems unreasonable, but in fact it is relatively consistent with the current distribution of children with disabilities by household registration in China. The occurrence of disability is caused by the combined effects of multiple factors such as natural conditions, medical and health care, living habits, and safety accidents. Due to natural factors such as inbred marriage, excessive fluoride content in drinking water, and lack of iodine in natural environment, rural communities are more prominent than urban communities; medical and health conditions such as medical conditions, disease prevention conditions, and the degree of medical and health knowledge diffusion, rural communities are worse than urban communities; alcohol and smoking among pregnant women, non-medical examination of pregnant women, wrong medication and drug stimulation during pregnancy, and accidents during delivery, rural communities are more than urban communities; consumption of junk food and Expired food, lack of nutrients, inadequate nutrition, improper nutritional mix and other life habits are more prominent in rural communities than in urban communities; traffic accidents, medical accidents, accidental injuries, carbon monoxide poisoning, drug poisoning, pesticide moderate, food poisoning and other life safety accidents are more likely in rural communities than in urban communities. These factors contribute to the fact that there are many more children with disabilities in rural than in urban areas. Therefore, in this study, after in-depth interviews and questionnaires in the rehabilitation institutions, the sample was mostly composed of rural children with disabilities.

Government support, institutional support, and family support all have significant and direct positive effects on the rehabilitation effects of disabled children. The richer the support resources available from the government, rehabilitation institutions, and families, the better the rehabilitation effects of disabled children. However, it is worth noting that the government is the leader, organizer, guide, and securer of the social support system for disabled children, and especially after the issuance of the rehabilitation assistance system for disabled children, the government is supposed to have a stronger direct influence on the rehabilitation of disabled children, however, in the table, the direct effect of government support on the rehabilitation effects of disabled children is significantly lower than that of institutional support and family support, which, to some extent, highlights that in promoting the rehabilitation of disabled children, the government still has certain shortcomings in system development rules, cooperation among departments, and public opinion publicity, lacks direct contact with disabled children and their families, and ignores the actual ideas of the demand side, leading to serious supply and demand deviation (1). Furthermore, the direct effect of institutional support is twice as large as the direct effect of family support, reflecting to some extent that the family-centered rehabilitation model is currently far less developed than institutional rehabilitation in China. Interestingly, the majority of studies in China and abroad have concluded that family-based rehabilitation is the mainstream trend for rehabilitation in the future. They believe that family-based rehabilitation not only has the advantages of being low-cost, efficient, sustainable and easy to implement, but also provides targeted and comprehensive rehabilitation services that are tailored to the individual needs of children with disabilities. Some scholars have also pointed out that family rehabilitation is a more effective alternative to institutional rehabilitation, as it is more effective than institutional rehabilitation in mobilizing confidence in rehabilitation and can achieve the same results as institutional rehabilitation at a lower cost (49, 50). So why did this result occur? The results bear some resemblance to Gelaw et al.'s view (51). Although family rehabilitation is theoretically superior to institutional rehabilitation, the development of a family rehabilitation model must be based on the optimisation of resources within the community and family in order to provide professional rehabilitation services. However, at present, the internal resources of Chinese families and communities are not only underdeveloped, but also face large urban-rural, regional and individual differences, making it impossible to provide efficient and professional rehabilitation services. Therefore, in the short term, institutional rehabilitation remains a necessary and effective option for the vast majority of disabled children in China, especially for those who require highly specialized and complex services.

Institutional support plays a partial mediating role between government support, family support, and disabled children's rehabilitation effects. Not only can government support and family support directly influence the rehabilitation effects of disabled children, but they can also indirectly influence the rehabilitation effects through institutional support, which effectively compensates for the loss of their direct effects and enables the three to initially form close interconnection. In the previous project-based social support system for the rehabilitation of disabled children, different subjects had clear boundaries, were disconnected and isolated, and input support resources for the rehabilitation of disabled children in a single linear manner (34). The above result shows that the rehabilitation assistance system, China's first special system for the rehabilitation of people with disabilities, has effectively built a preliminary bridge among different subjects with rehabilitation institutions as the medium, promoted the building process of the social support system for disabled children, and enabled the government, rehabilitation institutions, and families to interact in an integrated manner, to jointly promote the development of the rehabilitation of disabled children. However, it is worth noting that the effect of family support through institutional support on the rehabilitation effects is small, showing, to some extent, that families' expectations and supervision of institutions have not been fully effective, and the connection between family support and institutional support needs further deepening in the future. This finding is in line with some of the current international research findings. Theoretically, in-depth cooperation between families and institutions not only contributes to the specialization and comprehensiveness of the rehabilitation content, but also to the prolongation of the rehabilitation time, which has a positive effect on the effectiveness of rehabilitation for children with disabilities (52, 53). In practice, however, family members have difficulty engaging in dialogue with rehabilitation agencies due to labor shortages, high expectations of institutional rehabilitation, and a lack of willingness to participate (53, 54). Some Australian rehabilitation agencies have even attempted to increase family-caregiver contact time and strengthen collaboration by holding regular meetings with families, providing accommodation for families, offering care coordination support, using individualized care plans and involving families in shared decision-making (55). This suggests that the emphasis on strengthening the links between family support and institutional support and the integrated role of family support is a major focus of future research.

Government support, institutional support, and family support have non-equilibrium effects in promoting the rehabilitation effects of disabled children, among which, institutional support has the largest promoting effect on the rehabilitation effects of disabled children, followed by government support, and family support has the smallest promoting effect. The above results show that with the vigorous advancement of the rehabilitation of disabled children, the welfare effect of the rehabilitation assistance system for disabled children has emerged, the social support system for the rehabilitation assistance of disabled children is experiencing structural, social and kernel changes, the support from the subjects has evolved from the traditional closed and disconnected one-way resource support to the open, coordinated, and interactive multi-support, the delivery of resources between the subjects and the object has gradually evolved from the inherent “sending—receiving” single linear relationship to “sending—receiving—feedback—sending” two-way relationship, and a comprehensive and efficient interactive support system is gradually forming to advance the rehabilitation of disabled children, with families as the foundation, institutions as the main body, and the government as the core. However, it cannot be ignored that the sum of the total effects of government support and family support on the rehabilitation effects of disabled children is slightly greater than the total effect of institutional support on the rehabilitation effects of disabled children, which is slightly deviated from what Eskow and Nuri revealed about the effect of government support and Thomas revealed about the effect of family support (49, 56, 57). As the core of the rehabilitation system, the government is supposed to be slightly more effective than institutions, and as the foundation of the rehabilitation system, families are supposed to play a role similar to that of institutions, however, this paper shows that their actual effects are not as ideal as institutional support's effect. This highlights that institutional support should be the main force in building the rehabilitation assistance system for disabled children in the future and warns of the inadequate effects of government support and family support in improving the rehabilitation effects of disabled children, which are inhibited and need to be further strengthened.

### Policy recommendations

Based on the above, to further improve the rehabilitation effects of disabled children and promote the effective implementation of the rehabilitation assistance system, the following suggestions are hereby given:

In terms of further improving government support, it is important to increase support efforts and enhance the scientificity, effectiveness, and specificity of the support content. Specifically, efforts should be made to: (a) Firstly, strengthen the survey on the willingness of the demand side, enhance the specificity of system content revision, effectively distinguish the structure of rehabilitation needs of children of different ages, stages, and types of disabilities, sort out the total number of demanders for rehabilitation services, the number and percentage of demanders for various types of rehabilitation services, the types and percentage of services required by different disability classes, etc., and promote the transformation of inclusive rehabilitation to inclusive and targeted rehabilitation; (b) Secondly, strengthen the comprehensive cooperation and coordination among financial, health, human resources and social security, education, civil affairs, safety supervision, publicity, development and reform, and justice departments with DPFs as the core, to integrate administrative resources, form policy synergy, effectively ensure the effective connection among different welfare policies, jointly protect the rehabilitation rights and interests of disabled children, and reduce the heavy rehabilitation burden on disabled children and their families; and (c) Thirdly, strengthen the publicity and public opinion support of the system, and fully use internet self-media and the offline publicity channels of local governments at different levels in an organized, planned, and step-by-step manner to improve the clear awareness of the system among families of disabled children and society's care for and attention to disabled children.

In terms of further improving institutional support, it is important to optimize the comprehensive conditions of rehabilitation institutions and comprehensively enhance their rehabilitation treatment capacities. Specifically, efforts should be made to: (a) Firstly, raise the access threshold for designated rehabilitation institutions, strengthen the daily assessment of rehabilitation institutions, strictly eliminate unqualified institutions, urge rehabilitation institutions to independently optimize their environment and upgrade their rehabilitation equipment, and ensure the professional quality, completeness, and adaptability of the external environment such as sites, equipment, and space available from designated rehabilitation institutions; (b) Secondly, strengthen the specialization of rehabilitation personnel, build a new model of joint cultivation by universities and institutions, promote daily training in rehabilitation knowledge, and comprehensively enhance the comprehensive quality and abilities of rehabilitation personnel; (c) Thirdly, build a shared network of rehabilitation institutions, strengthen the sharing and connection of rehabilitation institutions, actively promote and popularize new rehabilitation techniques at home and abroad, and improve rehabilitation techniques and levels; and (d) Fourthly, promote the refined development of institutional rehabilitation departments, expand the service content of rehabilitation institutions, and urge institutions to comprehensively provide various scientific rehabilitation programs such as physical therapy, occupational therapy, speech therapy, hydrotherapy, prosthetics and orthotics, music therapy, psychotherapy, and social therapy.

In terms of improving family support, it is important to comprehensively increase family support through multiple channels. Specifically, efforts should be made to: (a) Firstly, comprehensively and effectively ensure the effective distribution of subsidies for low-income households, subsidies for people with disabilities, medical reimbursement funds, and special funds for the rehabilitation of disabled children, guide social financial assistance for families in difficulty, vigorously develop public welfare positions suitable for families with disabled children, and enhance the economic resilience of families with disabled children; (b) Secondly, improve the rehabilitation knowledge of other family members through multiple channels, provide targeted guidance on rehabilitation training, parent-child activities, daily maintenance of assistive devices, and other basic knowledge for families with different types of disabled children, and urge families to conduct daily and standardized basic rehabilitation activities; and (c) Thirdly, build a psychological aid system for parents, guide them to understand their children's disabilities objectively and scientifically, improve their confidence in treatment, and teach them basic knowledge of psychological guidance, so as to provide daily psychological counseling to disabled children and reduce the internal inferiority complex, loneliness, and social disengagement feeling of disabled children.

It is important to improve the social support system for the rehabilitation of disabled children, strengthen the effective connection between the subjects and between the subjects and object of social support, and build a comprehensive and efficient interactive support system with families as the foundation, institutions as the main body, and the government as the core. Specifically, efforts should be made to: (a) Firstly, strengthen the government's support for institutions, introduce reasonable and scientific preferential policies, change the funding method of allocating rehabilitation funds annually, reduce the survival pressure on rehabilitation institutions, and regularly conduct institution assessment and rehabilitation guidance, to effectively guarantee the service level and quality of rehabilitation institutions; (b) Secondly, strengthen the feedback on family needs to rehabilitation institutions, highlight families' supervision over institutions, and urge rehabilitation institutions to reasonably use rehabilitation funds, expand rehabilitation services, and provide accurate training in rehabilitation; and (c) Thirdly, completely break the traditional inherent “sending—receiving” linear delivery relationship between the subjects and the object, strengthen the forms of the demand side's expression of will through multiple channels, strengthen the connection channels between the subjects and the object, and create a regular “sending—receiving—feedback—sending” benign support relationship.

This study also has some limitations. Firstly, in terms of assessing the implementation effect of the system, because the rehabilitation assistance system for children with disabilities is a typical new thing, involving various fields such as rehabilitation medicine, sociology, and management, there is no mature method for assessing the implementation effect of the rehabilitation assistance system for children with disabilities in the academic field. As a result, the assessment of the effect of the implementation of the rehabilitation assistance system for children with disabilities is based only on the fundamental goal of the implementation of the rehabilitation assistance system for children with disabilities, and the effect of the rehabilitation of children with disabilities is selected as a direct variable to assess the effect of the implementation of the system, which may miss some information. The system is a macroscopic and systematic concept, and its implementation effects need to be measured from multiple dimensions. Therefore, in future studies on the implementation effects of the rehabilitation assistance system for children with disabilities, it is necessary to consider other indicators such as system coverage, accessibility, and equality, while taking into account the rehabilitation effects of children with disabilities. Secondly, in terms of the study population, the analysis was mainly conducted on a unified sample of urban children with disabilities and rural children with disabilities, and the differences in the implementation effects of rehabilitation assistance systems for children with disabilities by household registration were not explored in depth. The spatial heterogeneity and uniqueness of China's rehabilitation problems are determined by its vast territory and unbalanced development between urban and rural areas. Therefore, future studies should further refine the scope of the study to investigate the implementation effects of the rehabilitation assistance system for children with disabilities in rural and urban areas separately, and reveal and analyze the differences.

## Data availability statement

The raw data supporting the conclusions of this article will be made available by the authors, without undue reservation.

## Ethics statement

Ethical review and approval was not required for the study of human participants in accordance with the local legislation and institutional requirements. Written informed consent from the participants was not required to participate in this study in accordance with the national legislation and the institutional requirements.

## Author contributions

ZG: conceptualization, investigation, writing the original manuscript, revising the manuscript, and administration. HZ: conceptualization, investigation, writing the original manuscript, and revising the manuscript. HT: methodology, investigation, and writing the original manuscript. All authors contributed to the article and approved the submitted version.
